# Comparison of the trifecta outcomes of robotic and open nephron-sparing surgeries performed in the robotic era of a single institution

**DOI:** 10.1186/s40064-015-1274-2

**Published:** 2015-09-04

**Authors:** Ömer Acar, Esin Öztürk Işık, Tuna Mut, Yeşim Sağlıcan, Aslıhan Onay, Metin Vural, Ahmet Musaoğlu, Tarık Esen

**Affiliations:** 1 Department of Urology, School of Medicine, Koc University, Istanbul, Turkey; 2 Biomedical Engineering Institute, Boğaziçi University, Istanbul, Turkey; 3 Department of Urology, VKF American Hospital, Istanbul, Turkey; 4 Department of Pathology, School of Medicine, Acibadem University, Istanbul, Turkey; 5 Department of Radiology, School of Medicine, Koc University, Istanbul, Turkey; 6 Department of Radiology, VKF American Hospital, Istanbul, Turkey

**Keywords:** Kidney cancer, Robotics, Nephron-sparing surgery, Trifecta

## Abstract

**Purpose:**

In this study we aimed to report a comparative analysis between open and robotic nephron sparing surgeries (NSS) from a single institutional database.

**Methods:**

Patients who have undergone NSS during the robotic era of our institution were included in this study. Open (n = 74) and robotic (n = 59) groups were compared regarding trifecta outcome. Trifecta was defined as; warm ischemia time (WIT) <25 min, negative surgical margins and the absence of perioperative complications.

**Results:**

A total of 57 (77 %) and 45 (76 %) patients in the open and robotic groups, respectively achieved the trifecta outcome. Overall trifecta rate was 77 % (n = 102/133). The only statistically significant difference between trifecta positive and trifecta negative patients was the length of hospitalization (LOH). Except LOH; none of the tested parameters were shown to be predictive of trifecta outcome on univariate and multivariate analyses. Concerning trifecta positive patients; those in the open surgery group had larger tumors with a higher degree of morphometric complexity and were hospitalized for a longer period of time. Additionally, operative duration was significantly higher in the robotic group.

**Conclusions:**

In our cohort, no significant difference in achieving the trifecta outcome was reported after open and robotic NSS. Length of hospitalization was the only parameter that differed significantly between trifecta positive and trifecta negative patients. Surgical approach was not a significant predictor of simultaneous achievement of trifecta outcomes. Irrespective of the trifecta definition; larger and more complicated tumors were handled via open NSS.

## Background

The incidence of small renal masses has increased over the last decades due to the improvements and widespread use of imaging techniques (Kane et al. 2008). Nephron sparing surgery (NSS) is the gold standard treatment modality for cT1a renal tumors and it can be accomplished via open, laparoscopic and robot-assisted laparoscopic approaches. Moreover, whenever it is technically feasible, NSS has been advocated for the management of larger (≤7 cm) renal tumors (Ljungberg et al. 2010; Motzer et al. 2009). Robotic NSS has gained considerable popularity for the management of renal masses owing to its role in providing the advantages of minimally invasiveness while ensuring an easier transition from open surgery compared to pure laparoscopic surgery.

Trifecta has been adapted to describe the outcomes of patients undergoing robotic NSS. It’s initial definition that has been described by the University of Southern California Group includes negative surgical margins, no urologic complications, and a minimal decrease in renal function postoperatively. The same group reported that the range of patients achieving this outcome after robotic NSS was between 44 and 68 % (Hung et al. 2013; Khalifeh et al. 2013).

To compare open and robotic approaches, we herewith report a retrospective analysis of a single-surgeon series of ≤cT2a NSSs; evaluating clinical, surgical, pathologic, functional results and the simultaneous achievement of the trifecta outcomes.

## Patients and methods

This study includes all 133 patients who underwent NSS after the introduction of robotic technology in our institution (as of May 2010). All surgical procedures were carried out by a single surgeon (T.E.) who has accomplished a direct transition from open to robot-assisted surgery. Open (n = 74) and robotic (n = 59) groups were compared regarding clinical (age, A.S.A. score, tumor diameter, R.E.N.A.L. score, P.A.D.U.A. score and C-index), surgical (operative duration, estimated blood loss, length of hospitalization), functional (perioperative reduction in eGFR) results and trifecta outcome. Written informed consent was obtained from all patients prior to the retrospective chart review.

All patients demonstrated contrast-enhancing renal masses on preoperative computed tomography (CT) and/or magnetic resonance imaging (MRI). All treatment options were discussed thoroughly with the patients. Indications for NSS and technical details (open versus robotic NSS, off-clamp versus clamped NSS) were based on tumor characteristics (size, complexity, and location), patient comorbidities, and surgeon preference.

Tumor size was defined as the largest diameter of the tumor in cm. Based on preoperative radiologic findings, none of the patients had lymph node involvement or distant metastasis. Tumor morphometry was evaluated conjointly by two radiologists (M.V. and A.O. with 15-years and 4-years of experience in uroradiology, respectively) according to the R.E.N.A.L., P.A.D.U.A. and C-index methods (Kutikov and Uzzo 2009; Ficarra et al. 2009; Simmons et al. 2010). All surgical specimens were processed according to the standard pathologic procedures by a single pathologist (Y.S.) with a 10-years of experience in uropathology. Pathological data included histological subtype, T stage, Fuhrman grade and margin status. Tumor staging was designated according to the TNM classification based on the 2009 American Joint Committee on Cancer/International Union against Cancer Classification System.

All complications within 30 days of surgery were classified according to the modified Clavien system (Dindo et al. 2004). Serum creatinine was measured 1 day, 1 month, 3 months, and 6 months and then yearly after surgery. Estimated glomerular filtration rate (eGFR) was determined using the modified modification of diet in renal disease (MDRD) equation (Levey et al. 1999). Preoperative eGFR was compared to the eGFR on the last follow-up to assess long-term changes in renal function after NSS. Trifecta outcome was defined as a combination of warm ischemia time (WIT) <25 min, negative surgical margins and no perioperative complications (Hung et al. 2013; Khalifeh et al. 2013).

### Surgical technique

Open nephron-sparing surgery (ONSS) was performed using the intercostal (between 11th and 12th ribs) extraperitoneal flank approach, as previously described (Campbell and Novick 1995). Briefly, after adequate exposure of the kidney, Gerota’s fascia was opened and perinephritic fatty tissue was dissected off the renal surface. Ureter and the vascular pedicle were marked with vessel loops. The decision about hilar clamping was given perioperatively according to in situ findings and preoperative radiologic data. We did not implement cold-ischemia in any of these open NSSs. Tumors were removed via enucleoresection leaving a minimal margin of normal parenchyma (Kirkali and Canda 2008). Bleeding from the tumor bed was controlled with 3/0 polyglactin interrupted sutures and parenchyma was adapted with 2/0 monofilament running sutures, over a surgical bolster.

All robotic nephron-sparing surgeries (RNSS) were performed using the da Vinci Si surgical system (Intuitive Surgical, Inc., Sunnyvale, CA) with a 5-port approach, including two 8 mm ports for the robotic instruments, one 12 mm port for the robotic scope, and 2 ports for the bedside assistant. RNSS’s were carried out through the transperitoneal route with the patient in flank position. After colonic mobilization, Gerota’s fascia was opened and tumor was adequately exposed. The decision to clamp renal pedicle was given during the operation, based on CT and/or MR images and intraoperative findings. If there was such a need, the renal artery was occluded with an external vessel loop secured with a hem-o-lok clip over a silicone tube (Rassweiler et al. 2000). After demarcating tumor margins with electrocautery, resection was carried out using cold-scissors. All tumors were enucleoresected leaving a minimal margin of normal parenchyma. Tumor bed was oversewn with 3/0 polyglactin sutures (in case of pelvicalyceal violation) and parenchyma was approximated using the “sliding clip” technique (Benway et al. 2009).

### Statistical analysis

All statistical analyses were two-sided and performed using Statistics Toolbox within MATLAB (The Mathworks Inc., Natick, MA). A Mann–Whitney ranksum test was utilized to determine statistically significant parameter differences between the trifecta positive and trifecta negative patients, and between the trifecta positive patiens of the open and robotic NSS groups. The categorical variables were compared between robotic and open surgery subgroups with a Chi square test for proportions. The predictive factors for a positive trifecta outcome were identified by univariate and multivariate analyses using the Cox proportional hazards regression model. The differences were considered as statistically significant at a p value of <0.05.

## Results

Clinical, surgical and functional results of all the open and robotic NSSs are summarized in Table 1. A total of 57 (77 %) and 45 (76 %) patients in the open and robotic groups, respectively achieved the trifecta outcome and these values were not significantly different from each other. Overall trifecta rate was 77 % (n = 102/133). The reason for the failure to achieve trifecta was; the presence of complications (n = 15), surgical margin positivity (n = 1) and prolonged WIT (44 min) in addition to the presence of complications (n = 1) in the open NSS group. On the other hand, complications (n = 9), prolonged WIT (n = 3, mean value of 31.6 min), surgical margin positivity in addition to the presence of complications (n = 1) and prolonged WIT (36 min) in addition to the presence of complications (n = 1) constituted the causes of trifecta negativity in the robotic NSS group.

**Table 1:** The differences between open and robotic NSS groups in terms of clinical, surgical and functional results

Parameter	Open (n = 74)	Robotic (n = 59)	*p* value
Mean patient age (years)	55.3 ± 11.2	51 ± 13.3	*0.04*
Gender (M/F)	48/26	46/13	0.0992
Mean A.S.A. score	1.6 ± 0.7	1.5 ± 0.6	0.4
Mean R.E.N.A.L. score	7.6 ± 1.9	6.1 ± 1.7	*0.0001*
Mean P.A.D.U.A. score	8.5 ± 1.7	7.3 ± 1.5	*0.0001*
Mean C-index value	1.3 ± 0.6	1.5 ± 0.4	0.1
Operated side (R/L)	32/42	25/34	0.9197
Mean operative duration (min)	101.01 ± 32.1	143.7 ± 46.5	*0.0001*
Mean estimated blood loss amount (ml)	187.2 ± 128.5	201.1 ± 223.4	0.6
Mean WIT (min)	7.6 ± 9.5	8.3 ± 11.4	0.72
Mean pathological tumor diameter (cm)	4.2 ± 2.02	3.4 ± 2.4	*0.04*
Mean length of hospitalization (days)	4.3 ± 1.7	3.8 ± 1.2	*0.02*
Mean preoperative eGFR (ml/min/1.73 m^2^)	85.02 ± 22.2	89.8 ± 16.6	0.1
Mean postoperative eGFR (ml/min/1.73 m^2^)	76.1 ± 20.9	83.8 ± 16.6	*0.02*
Mean perioperative reduction in eGFR (ml/min/1.73 m^2^)	−8.90 ± 16.13	−6.01 ± 11.09	0.49
Complications, Clavien Grade: 2/3a/3b/4 (n)	15/5/5/1	13/2/2/0	0.5904

Italic values indicate statistical significance

A total of 26 and 17 Clavien grade ≥2 complications were recorded in 16 and 11 patients in the open and robotic NSS groups, respectively. The distribution of the complication grades are listed in Table 1.

While analyzing the study group as a whole, the only statistically significant difference between trifecta positive and trifecta negative patients was the length of hospitalization (LOH) (Table 2). Similarly, LOH was the only parameter that differed significantly between trifecta positive and trifecta negative patients, both in the open and in the robotic subgroups. On univariate and multivariate analyses, none of the parameters (including surgical approach) but only LOH was shown to be predictive of trifecta positivity in general (Table 3). The findings were the same after repeating the analyses in open surgery (Table 4) and robotic surgery subgroups (Table 5).

**Table 2:** The differences between trifecta positive and trifecta negative NSSs (open and robotic) in terms of clinical, surgical and functional results

All NSSs (n = 133) % trifecta = 0.77	Trifecta = 1 (n = 102)	Trifecta = 0 (n = 31)	*p* value
Mean patient age (years)	53.2 ± 12.7	53.8 ± 11.3	0.89
Mean A.S.A. Score	1.5 ± 0.6	1.6 ± 0.7	0.45
Mean R.E.N.A.L. score	6.9 ± 2.0	6.8 ± 1.9	0.75
Mean P.A.D.U.A. score	8.03 ± 1.7	8.0 ± 1.6	0.94
Mean C-index value	1.4 ± 0.6	1.3 ± 0.4	0.30
Mean pathological tumor diameter (cm)	3.8 ± 2.3	3.8 ± 1.7	0.48
Mean operative duration (min)	116.9 ± 41.03	129.8 ± 53.8	0.27
Mean estimated blood loss amount (ml)	169.2 ± 104.5	272.9 ± 302.5	0.11
Mean length of hospitalization (days)	3.7 ± 0.9	5.4 ± 2.1	*1.2* *×* *10* ^*−6*^
Perioperative reduction in eGFR (ml/min/1.73m2)	−7.68 ± 14.05	−7.40 ± 14.69	0.74

Italic value indicates statistical significance

**Table 3:** Univariable and multivariable Cox analyses were performed to identify variables predictive of trifecta positive outcomes in the whole study population (including patients who have undergone open and robotic NSS)

Covariate	Univariate analysis			Multivariate analysis		
	Coefficient (bi)	HR [exp(bi)]	*p* value	Coefficient (bi)	HR [exp(bi)]	*p* value
Surgical approach (open/robotic)	0.0076	1.0076	0.9654	0.1113	1.1177	0.6466
Mean patient age	0.0006	1.0006	0.9289	−0.0027	0.9973	0.7715
Gender	−0.0747	0.9280	0.6949	−0.0164	0.9837	0.9377
Mean A.S.A. score	0.0419	1.0427	0.7440	−0.0814	0.9218	0.6446
Mean R.E.N.A.L. score	−0.0068	0.9932	0.8753	−0.0515	0.9498	0.6174
Mean P.A.D.U.A. score	−0.0018	0.9982	0.9721	−0.0055	0.9946	0.9608
Mean C-index value	−0.0448	0.9562	0.7592	−0.1021	0.9030	0.6169
Mean operative duration	0.0013	1.0013	0.5238	−0.0017	0.9983	0.5065
Mean estimated blood loss amount	0.0009	1.0009	0.0846	0.0010	1.0010	0.1418
Mean WIT	0.0096	1.0097	0.2819	0.0145	1.0146	0.1510
Mean pathological tumor diameter	−0.0019	0.9981	0.9593	0.0094	1.0095	0.8368
Mean length of hospitalization	0.1827	1.2005	*0.0012*	0.2259	1.2534	*0.0013*
Mean perioperative reduction in eGFR	0.0003	1.0003	0.9662	0.0039	1.0039	0.5733

Except length of hospital stay; none of the tested parameters were shown to be predictive of trifecta positivity

Italic values indicate statistical significance

**Table 4:** Univariable and multivariable Cox analyses were performed to identify variables predictive of trifecta positive outcomes in the open surgery group

Covariate	Univariate analysis			Multivariate analysis		
	Coefficient (bi)	HR [exp(bi)]	*p* value	Coefficient (bi)	HR [exp(bi)]	*p* value
Mean patient age	0.0013	1.0013	0.8945	−0.0034	0.9966	0.8220
Gender	−0.1152	0.8912	0.6362	−0.1928	0.8247	0.4899
Mean A.S.A. score	0.0449	1.0460	0.7834	−0.1564	0.8552	0.5204
Mean R.E.N.A.L. score	−0.0068	0.9933	0.9122	−0.0808	0.9224	0.5453
Mean P.A.D.U.A. score	0.0117	1.0118	0.8617	0.0450	1.0460	0.7320
Mean C-index value	−0.0777	0.9253	0.6480	−0.1456	0.8645	0.5655
Mean operative duration	0.0007	1.0007	0.8592	−0.0032	0.9968	0.4960
Mean estimated blood loss amount	0.0008	1.0008	0.3978	0.0009	1.0009	0.4700
Mean WIT	0.0085	1.0086	0.5145	0.0110	1.0111	0.4832
Mean pathological tumor diameter	0.0101	1.0102	0.8569	0.0165	1.0166	0.7815
Mean length of hospitalization	0.1641	1.1783	*0.0169*	0.2121	1.2363	*0.0193*
Mean perioperative reduction in eGFR	0.0017	1.0017	0.8187	0.0073	1.0074	0.4583

Except length of hospital stay; none of the tested parameters were shown to be predictive of trifecta positivity

Italic values indicate statistical significance

**Table 5:** Univariable and multivariable Cox analyses were performed to identify variables predictive of trifecta positive outcomes in the robotic surgery group

Covariate	Univariate analysis			Multivariate analysis		
	Coefficient (bi)	HR [exp(bi)]	*p* value	Coefficient (bi)	HR [exp(bi)]	*p* value
Mean patient age	0.0001	1.0001	0.9923	−0.0002	0.9998	0.9904
Gender	−0.0083	0.9917	0.9788	0.1756	1.1919	0.6369
Mean A.S.A. score	0.0385	1.0393	0.8531	0.0153	1.0154	0.9568
Mean R.E.N.A.L. score	−0.0078	0.9923	0.9165	0.0151	1.0152	0.9356
Mean P.A.D.U.A. score	−0.0218	0.9784	0.7997	−0.1063	0.8991	0.6374
Mean C-index value	0.0538	1.0553	0.8590	−0.1258	0.8818	0.7563
Mean operative duration	0.0022	1.0022	0.4514	−0.0025	0.9975	0.5106
Mean estimated blood loss amount	0.0010	1.0010	0.1246	0.0013	1.0013	0.1014
Mean WIT	0.0106	1.0107	0.3885	0.0174	1.0175	0.2279
Mean pathological tumor diameter	−0.0110	0.9891	0.8304	0.0032	1.0032	0.9713
Mean length of hospitalization	0.3063	1.3584	*0.0143*	0.3830	1.4667	*0.0132*
Mean perioperative reduction in eGFR	−0.0035	0.9965	0.7682	−0.0024	0.9976	0.8645

Except length of hospital stay; none of the tested parameters were shown to be predictive of trifecta positivity

Italic values indicate statistical significance

Concerning the comparison between trifecta positive patients in the open and robotic groups (Table 6); those in the open surgery group had larger tumors with higher R.E.N.A.L. and P.A.D.U.A. scores and were hospitalized for a longer period of time. Additionally, operative duration was significantly higher in the robotic group when compared with that recorded in the open group.

**Table 6:** The differences between trifecta positive patients in open and robotic NSS groups in terms of clinical, surgical and functional results

Parameter	Open (n = 57)	Robotic (n = 45)	*p* value
Mean patient age (years)	55.12 ± 11.59	50.98 ± 13.79	0.12
Mean A.S.A. score	1.58 ± 0.71	1.49 ± 0.63	0.59
Mean R.E.N.A.L. score	7.67 ± 1.91	6.13 ± 1.79	*0.0001*
Mean P.A.D.U.A. score	8.51 ± 1.74	7.42 ± 1.56	*0.0015*
Mean C-index value	1.43 ± 0.72	1.54 ± 0.43	0.22
Mean operative duration (min)	100.18 ± 31.40	138.20 ± 42.24	*2.3* *×* *10* ^*−6*^
Mean estimated blood loss amount (ml)	172.54 ± 111.92	165.11 ± 95.57	0.69
Mean pathological tumor diameter (cm)	4.15 ± 2.07	3.50 ± 2.74	*0.005*
Mean length of hospitalization (days)	3.96 ± 1.02	3.44 ± 0.76	*0.0003*
Mean perioperative reduction in eGFR (ml/min/1.73 m^2^)	−9.44 ± 15.84	−5.46 ± 11.16	0.22

Italic values indicate statistical significance

The difference between trifecta positive and trifecta negative patients in terms of the reduction of eGFR at 6-month follow-up was not statistically significant. Similarly, when open and robotic groups were compared as a whole, the eGFR change from baseline was similar.

## Discussion

Curing the cancer, saving the kidney and utilizing minimally invasive approaches when technically feasible can be considered as the surgical priorities in the management of renal masses (Coffin et al. 2011). However, postoperative complications are also of utmost importance since intraoperative and postoperative problems may necessitate additional interventions, prolonged hospitalization, and may lead to morbidity and even mortality (Laviana and Hu 2014). The term “trifecta”, which addresses the surgical margin status, duration of warm-ischemia and the presence of complications, was introduced in an effort to standardize the way of reporting NSS success and was initially used for robotic surgeries (Hung et al. 2013).

Khalifeh et al. reported a 31.6 % trifecta rate in a large single-surgeon pure laparoscopic NSS series and also noted that trifecta achievement is much more probable in robotic NSS (59 %) when compared to the pure laparoscopic approach (Khalifeh et al. 2013). Their relatively low trifecta rate may be explained by; the technically demanding nature of the initial laparoscopic NSSs and the different inclusion criteria used. Hung et al. reported the trifecta achievement rate in a contemporary series consisting of NSSs for cT1 renal masses as 68 %. However, they defined trifecta as; negative surgical margin, minimal renal functional decrease and no urological complication (Hung et al. 2013).

On the other hand, in their recent matched-pair comparative analysis consisting of over 400 patients with clinical T1a renal masses, Minervini et al. reported the trifecta rate as 78.6 and 74.3 % in open and laparoscopic NSS, respectively. Moreover, the surgical approach was not a significant predictor of a negative trifecta on multivariable analysis (Minervini et al. 2014). In our study, we have found similar results and the rate of simultaneous achievement of the trifecta outcomes did not differ significantly between open (77 %) and robotic (76 %) NSS groups. Likewise, surgical approach was not a significant predictor of trifecta positivity in our series.

The trifecta positivity rate in our cohort does not seem to be affected by the outcome of the robotic NSSs done in the early phase of the learning curve. The difference between the initial (between May 2010 and September 2012) and latter (between September 2012 and December 2014) NSSs in terms of the trifecta achievement rate was statistically insignificant both in the open (88.4 % and 70.8 %, respectively) and robotic (83.3 % and 65.2 %, respectively) surgery groups. This can be the result of surgeon expertise level as proficient open surgeons require less knowledge for a successfull transition to robotic surgery compared to novice open surgeons (Sood et al. 2015).

Despite the fact that the majority of perioperative complications were of Clavien grade 2 both in the open and robotic NSS groups, their sole presence was the main factor underlying trifecta negativity in our cohort. Furthermore, the distribution of complication severity was similar between the open and robotic NSS groups. A total of 7 patients (2 in the open NSS and 5 in the robotic NSS group) could not achieve trifecta due to the either surgical margin positivity and/or prolonged WIT.

Length of hospitalization, which is expected to be prolonged due to a complicated postoperative course, was the only parameter that showed a statistically significant difference between trifecta positive and trifecta negative patients. Moreover, LOH was shown to be the only parameter that can predict trifecta outcome in our study population. These findings are quite understandable since per definition that trifecta necessitates the absence of complications. Undoubtedly, if the absence of urological complications is used as a criterion (Hung et al. 2013) instead of the virtual absence of any kind of complication, then the trifecta rate would have been higher in our series.

When open and robotic NSS groups were compared without applying the trifecta criteria; patient age, R.E.N.A.L. score, P.A.D.U.A. score, excised tumor diameter and length of hospitalization were found to be significantly higher in the open surgery group while operative duration being significantly longer in the robotic surgery group. Likewise, when the trifecta achievers in the open and robotic NSS groups were compared; the results were almost the same except for a statistically insignificant age difference. Nevertheless, the conclusion that can be drawn from this evaluation does not change, as patients with larger tumors with a more complicated morphometric profile, hence with an increased likelihood of perioperative complications, were managed via open NSS and inevitably they stayed in the hospital for a longer period of time than their robotic counterparts.

In the landmark study conducted by Thompson et al., the threshold WIT, above which the risk of postoperative acute kidney injury and de-novo stage 4 chronic kidney disease increases significantly, has been reported to be 25 min (Thompson et al. 2010). However, this observation was based on the functional outcome of NSSs performed on solitary kidneys and subsequent studies documented that WIT should be limited to 20 min irrespective of the surgical approach (Thompson et al. 2010; Nguyen and Gill 2008; Lane et al. 2013). In our series, less than 50 % of the patients in the robotic (45.9 %, n = 34) and open (37.2 %, n = 22) NSS groups were managed with the mean WIT being 16.7 and 22.3 min, respectively. Those who have exceeded the limit of 25 min in our cohort (n = 5) had a complicated tumor morphometry (mean R.E.N.A.L. score = 7.2, mean P.A.D.U.A. score = 8.6, mean C-index value = 1.3) and mean WIT in this small subgroup was 34.4 min (range = 26–44 min). Additionally, the postoperative eGFR change at 6-month follow-up was similar between open and robotic NSS groups.

A negative surgical margin does not guarentee a recurrence-free follow-up period and similarly the absence of urological complications, which has been used as a component of trifecta in other series, may not mean anything for a patient who has been hospitalized longer due to a non-urological problem. Likewise, even a limited duration of warm-ischemia (<25 min) can lead to adverse functional outcomes, particularly if the indication for NSS is an absolute one (Thompson et al. 2010). Although the achievement of trifecta may be translated as an early indication of surgical success, long-term follow-up is needed to validate the actual value of these criteria. Therefore the ideal way of reporting success in NSS series may be in the form of pentafecta, which includes long-term stability of preoperative renal function, recurrence-free follow-up, and complication-free perioperative period in addition to trifecta (Krane and Hemal 2014).

The main drawback of our study is the retrospective study design with its inherent selection biases. A proper randomization between open and robotic NSS would have served better to confirm the superiority of one approach over the other. Moreover, renal function was evaluated with the MDRD formula and not the nuclear imaging studies which could have been a more accurate way of determining function in each renal unit.

## **Conclusions**

In our cohort, the rate of simultaneous achievement of the trifecta outcomes was similar between open and robotic NSS groups. The presence of complications was the main reason for trifecta negativity. Except for the length of hospital stay, all of the tested parameters were comparable between trifecta positive and trifecta negative patients. Surgical approach was not a significant predictor of trifecta positivity. Irrespective of the trifecta definition; open NSS was associated with larger tumor size, more complicated tumor morphometry and longer length of hospital stay.
