# Erratum to: Comparison of the trifecta outcomes of robotic and open nephron-sparing surgeries performed in the robotic era of a single institution

**DOI:** 10.1186/s40064-015-1450-4

**Published:** 2015-11-02

**Authors:** Ömer Acar, Esin Ozturk-Isik, Tuna Mut, Yeşim Sağlıcan, Aslıhan Onay, Metin Vural, Ahmet Musaoğlu, Tarık Esen

**Affiliations:** Department of Urology, School of Medicine, Koc University, Istanbul, Turkey; Biomedical Engineering Institute, Boğaziçi University, Istanbul, Turkey; Department of Urology, VKF American Hospital, Istanbul, Turkey; Department of Pathology, School of Medicine, Acibadem University, Istanbul, Turkey; Department of Radiology, School of Medicine, Koc University, Istanbul, Turkey; Department of Radiology, VKF American Hospital, Istanbul, Turkey

## Erratum to: SpringerPlus (2015) 4:472 DOI 10.1186/s40064-015-1274-2

It has come to our attention that during the production of the original article (Acar et al. 2015), the name of the second author, Esin Ozturk-Isik, was spelt as Esin Öztürk Işık, which is incorrect and differs from how her name appears in previously published work. The publisher apologises for any inconvenience caused by this.
